# Superhydrophobicity in perfection: the outstanding properties of the lotus leaf

**DOI:** 10.3762/bjnano.2.19

**Published:** 2011-03-10

**Authors:** Hans J Ensikat, Petra Ditsche-Kuru, Christoph Neinhuis, Wilhelm Barthlott

**Affiliations:** 1 Nees Institute, University of Bonn, Meckenheimer Allee 170, 53115 Bonn, Germany; 2 Institut für Botanik, Technische Universität Dresden, Zellescher Weg 20b, 01069 Dresden, Germany

**Keywords:** epicuticular wax, leaf surface, Lotus effect, papillae, water repellency

## Abstract

Lotus leaves have become an icon for superhydrophobicity and self-cleaning surfaces, and have led to the concept of the ‘Lotus effect’. Although many other plants have superhydrophobic surfaces with almost similar contact angles, the lotus shows better stability and perfection of its water repellency. Here, we compare the relevant properties such as the micro- and nano-structure, the chemical composition of the waxes and the mechanical properties of lotus with its competitors. It soon becomes obvious that the upper epidermis of the lotus leaf has developed some unrivaled optimizations. The extraordinary shape and the density of the papillae are the basis for the extremely reduced contact area between surface and water drops. The exceptional dense layer of very small epicuticular wax tubules is a result of their unique chemical composition. The mechanical robustness of the papillae and the wax tubules reduce damage and are the basis for the perfection and durability of the water repellency. A reason for the optimization, particularly of the upper side of the lotus leaf, can be deduced from the fact that the stomata are located in the upper epidermis. Here, the impact of rain and contamination is higher than on the lower epidermis. The lotus plant has successfully developed an excellent protection for this delicate epistomatic surface of its leaves.

## Introduction

**Figure 1:**
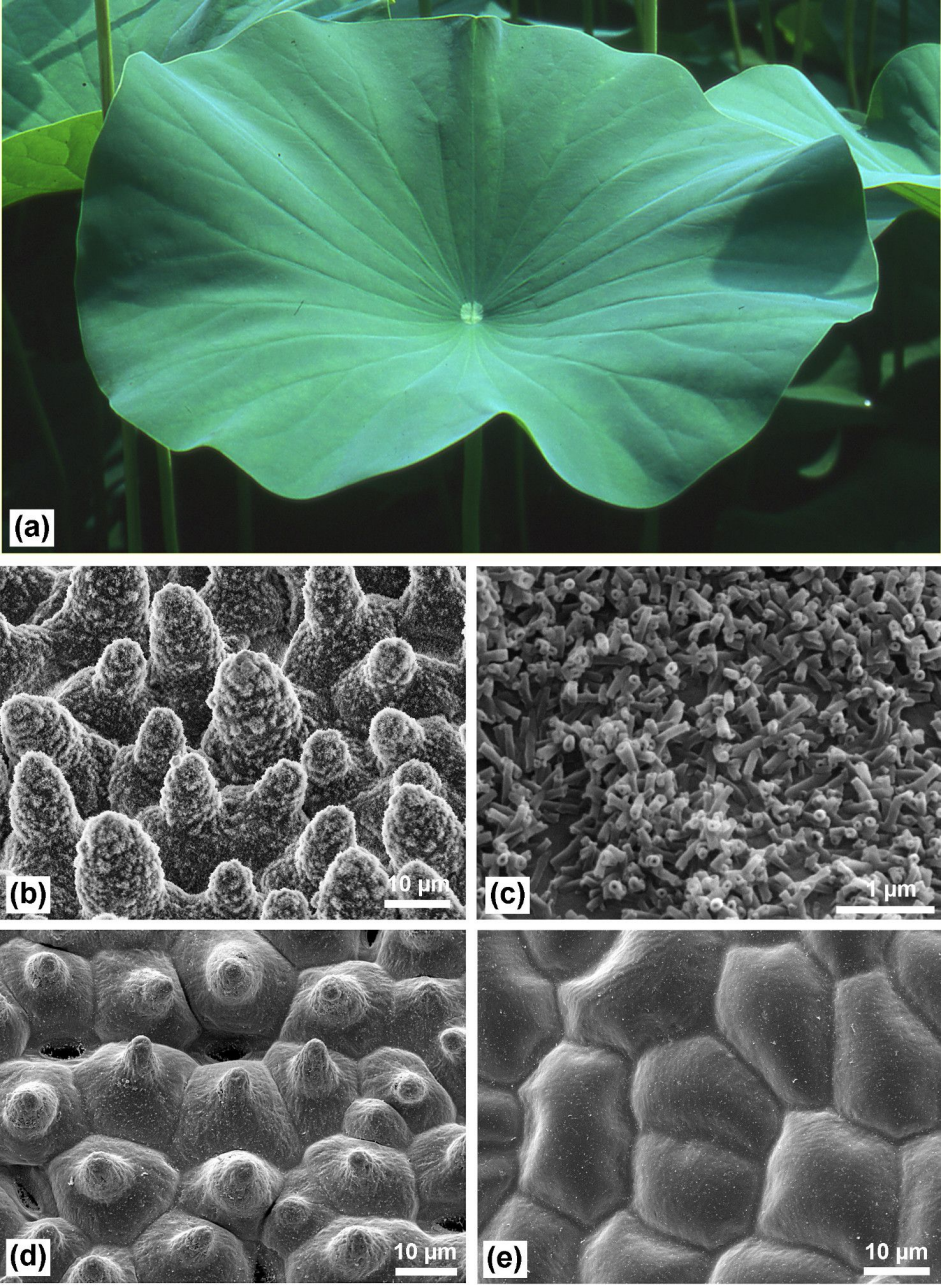
(a) Lotus leaves, which exhibit extraordinary water repellency on their upper side. (b) Scanning electron microscopy (SEM) image of the upper leaf side prepared by ‘glycerol substitution’ shows the hierarchical surface structure consisting of papillae, wax clusters and wax tubules. (c) Wax tubules on the upper leaf side. (d) Upper leaf side after critical-point (CP) drying. The wax tubules are dissolved, thus the stomata are more visible. Tilt angle 15°. (e) Leaf underside (CP dried) shows convex cells without stomata.

Since the introduction of the ‘Lotus concept’ in 1992 [1–2], the lotus leaf became the archetype for superhydrophobicity and self-cleaning properties of plant surfaces and a model for technical analogues [3–4] . Lotus (*Nelumbo nucifera*) is a semi-aquatic plant and develops peltate leaves up to 30 cm in diameter with remarkable water repellency. As an adaptation to the aquatic environment – some of the leaves float occasionally on the water surface – the stomata are located in the upper epidermis. The lower epidermis consists of convex cells covered with wax tubules and contains only few stomata. The upper epidermis features the distinctive hierarchical structure consisting of papillae with a dense coating of agglomerated wax tubules, which is the basis for the famous superhydrophobicity (Figure 1).

However, a hierarchical surface structure which induces strong water repellency and contact angles above 150° is not a special feature of lotus leaves. It has been known for a long time that plant surfaces covered with epicuticular wax crystals are water repellent, and that this feature is enhanced when the epidermis has additional structures such as papillae or hairs [5–6]. Neinhuis and Barthlott (1997) [7] presented an overview of more than 200 species with contact angles >150° and their surface morphologies. Many studies, in which the properties of lotus leaves were compared with those of other superhydrophobic plants, have shown the superiority of the upper side of the lotus leaf. A standard tool for the determination of wettability or water repellency is the measurement of the static contact angle by the ‘sessile drop’ method. Neinhuis and Barthlott (1997) [7] for example, measured contact angles on the lotus leaf of 162°, which are among the highest of the compared species, but many other (43%) of the tested superhydrophobic plants also showed contact angles between 160 and 163°. Even some species with flat epidermis cells but with a dense layer of epicuticular wax crystals, such as *Brassica oleracea* or some *Eucalyptus* species, can exhibit contact angles >160°. Thus, the contact angle alone is not suitable for a differentiated comparison of superhydrophobic samples. Other values such as contact angle hysteresis or roll-off (tilting) angle show more clearly the differences between the species. Mockenhaupt et al. (2008) [8] compared the tilting angles and the stability of the superhydrophobicity of various plants under moisture condensation conditions. Only the lotus leaves showed no significant loss of water repellency when water vapour condensed on the surface of the cooled samples at 5 °C. Wagner et al. (2003) [9] examined the morphology of the epidermal structures and the wettability with liquids of varying surface tension such as methanol–water mixtures. They reported the lowest wettability by these liquids for the lotus leaves in comparison to other species. They also described the unique shape of the papillae and a very high papillae density (number per area). Chemical analyses [10] and crystal structure analysis by X-ray diffraction [11] showed unique properties of the epicuticular wax of the lotus. The high content of nonacosanediols leads to a high melting point as well as a strongly disturbed crystal structure which is the basis for the formation of tubules. The visualization of the contact zone between leaves and droplets with cryo-scanning electron microscopy demonstrated the extremely reduced contact area for lotus [12]. Zhang et al. (2008) [13] made detailed measurements of the water repellency of the papillose lotus leaf surface in comparison with the non-papillose leaf margin. The importance of the nanoscopic wax crystals for the water repellency was demonstrated by Cheng et al. (2006) [14]. They reported a strong decrease of the contact angle after melting of the waxes. A limited air retaining capability of submersed lotus leaves was reported by Zhang et al. (2009) [15] after the leaves were held at a depth of 50 cm for 2 h. Bhushan et al. (2010) [4] used the surface structures of the lotus leaf as model for the development of artificial biomimetic superhydrophobic structures.

It became obvious that the outstanding and stable superhydrophobicity of the lotus leaf relies on the combination of optimized features such as the surface topography, robustness and the unique properties of the epicuticular wax. The aim of this article is to integrate the relevant features of the lotus leaf, and to compare them with superhydrophobic leaves of other plant species in order to illustrate their significance.

## Results and Discussion

### The properties of the lotus leaves

The lotus leaf shows an outstanding water repellency particularly on its upper (adaxial) side, which is more robust and less sensitive to mechanical damage than the under (abaxial) side. The reasons for these superior properties can be ascribed to the combination of micro- and nano-structures with optimized geometry and the unique chemical composition of the epicuticular waxes. These properties are illustrated in the following sections and compared with those of other superhydrophobic leaves. (The species are listed in the Experimental section).

**Figure 2:**
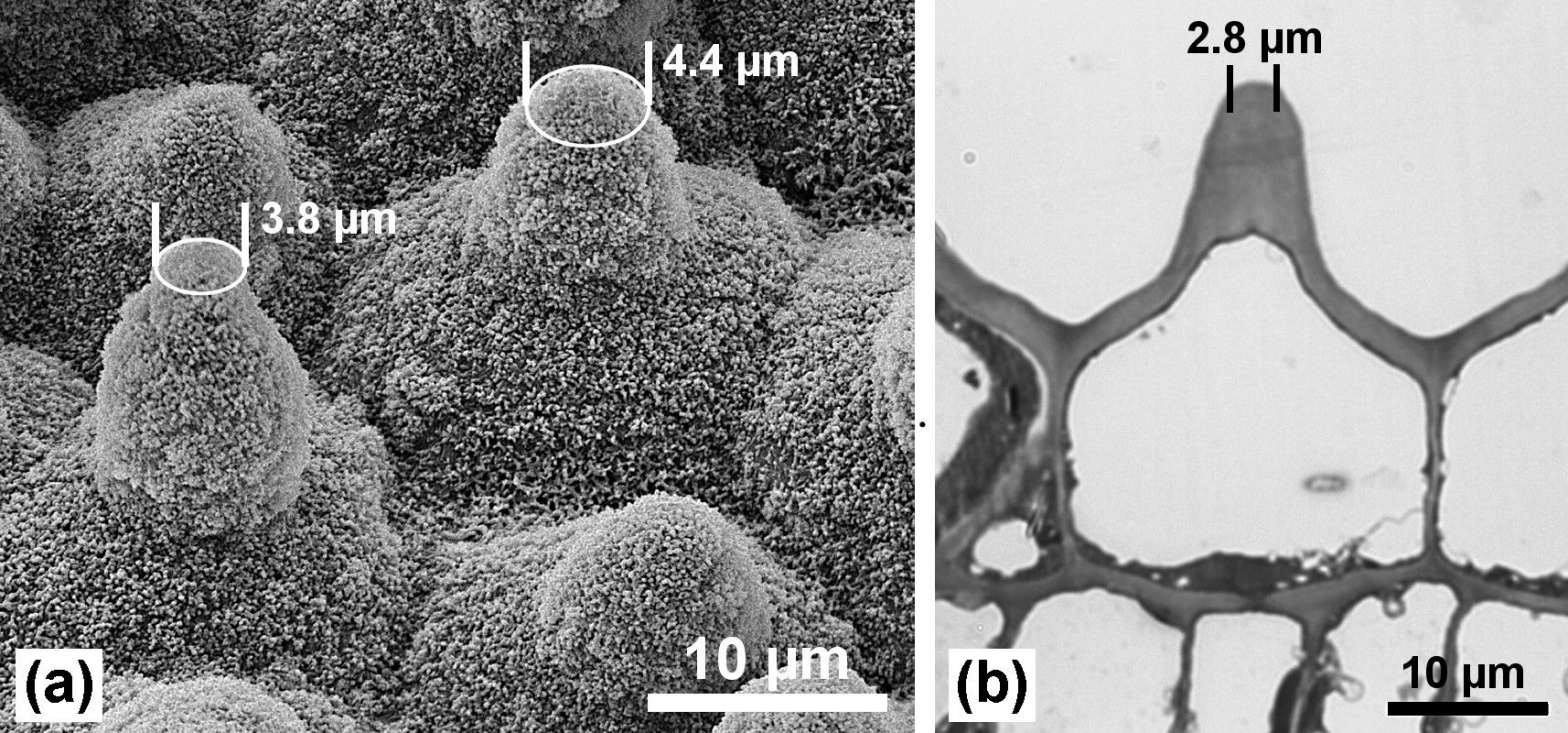
Epidermis cells of the leaf upper side with papillae. The surface is densely covered with wax tubules. (a) SEM image after freeze drying. (b) Light microscopy (LM) image of thin section of an embedded sample. Assuming a contact angle of >140°, for example, the area of heterogeneous contact between single papillae and water (marks) is small in comparison to the epidermis cell area.

**Minimization of the water-to-leaf contact area:** The epidermis cells of the upper leaf side form papillae of varying height and with a unique shape. The diameter of the papillae is much smaller than that of the epidermis cells and each papilla apex is not spherical but forms an ogive (Figure 2).

The whole surface is covered with short wax tubules which often accumulate in clusters. In comparison with other papillose plant surfaces, lotus has the highest density of papillae, but the lotus papillae have much smaller diameters which reduces the contact area with water drops; strictly speaking, the area of heterogeneous contact between surface and water. The contact area depends on the hydrophobicity of the surface and on the pressure of the water or on the kinetic energy or velocity of the striking water drops. At low pressures, caused by resting or rolling water droplets, the contact area is determined by the local contact angle of the surface structures. For the surface of a papilla coated with wax tubules, a superhydrophobic behavior with a local contact angle of >140° can be assumed. So, the diameter of the contact areas can be estimated from the SEM images and the cross sections of the selected samples (Figure 2).

**Figure 3:**
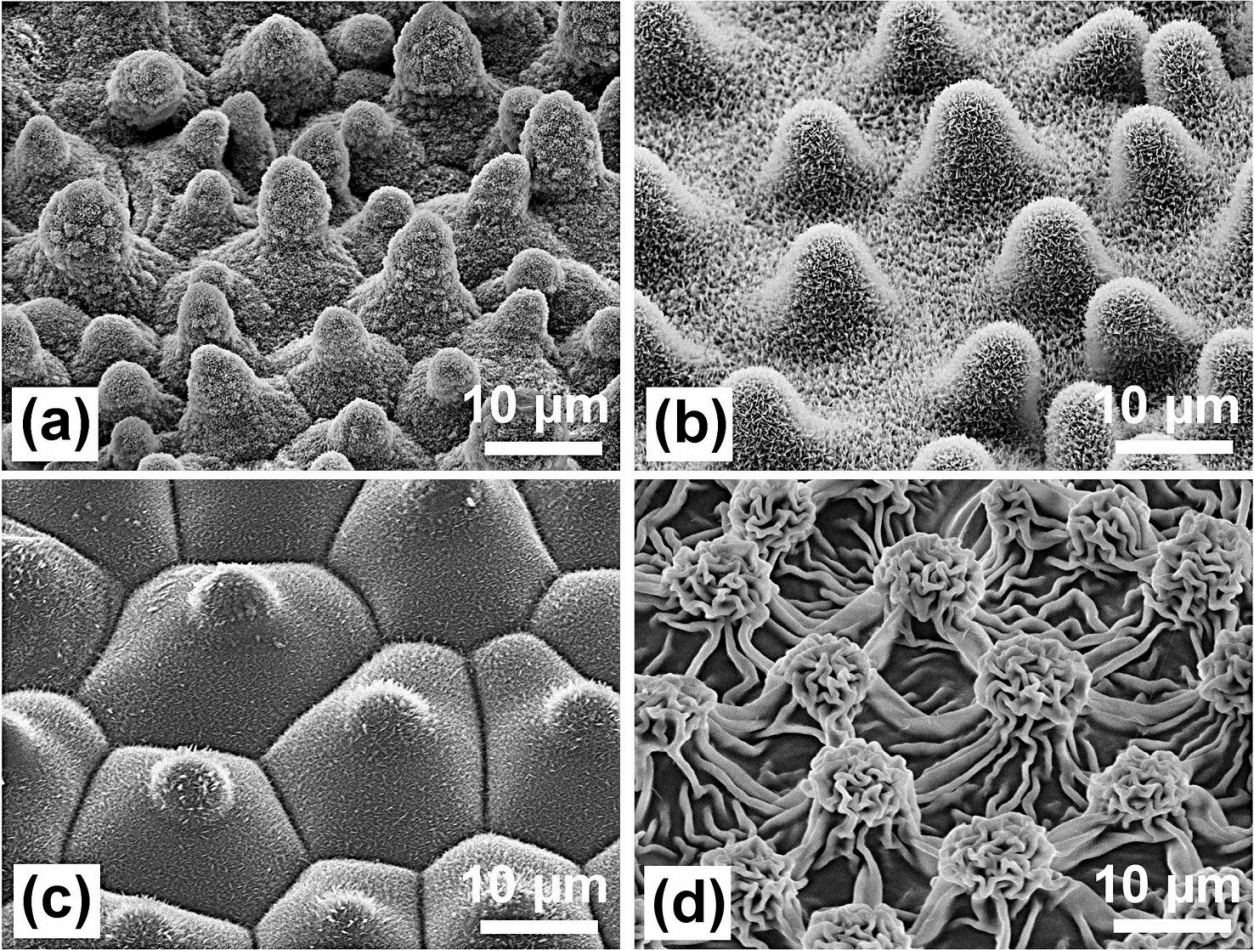
SEM images of the papillose leaf surfaces of *Nelumbo nucifera* (Lotus) (a), *Euphorbia myrsinites* (b), *Colocasia esculenta* (c), and *Alocasia macrorrhiza* (d). Lotus has the highest density of papillae with varying heights and the smallest diameter of the papillae. The papillae of the other species have larger diameters and are covered with different wax types: wax platelets *(E. myrsinites* and *C. esculenta*) and a wax film (*A. macrorrhiza)* which covers cuticular foldings.

The minimized contact area is the basic cause for the very low adhesion of water and, thus, the small roll-off (tilting) angles. Compared with lotus, the papillae on the leaves of the other plants (*E. myrsinites*, *C. esculenta*, *A. macrorrhiza*) (Figure 3, see also Figure 7) have much larger diameters and tip radii, and are covered with different wax types, wax platelets or wax film, respectively, which have a lower water repellency than wax tubules.

**Figure 4:**
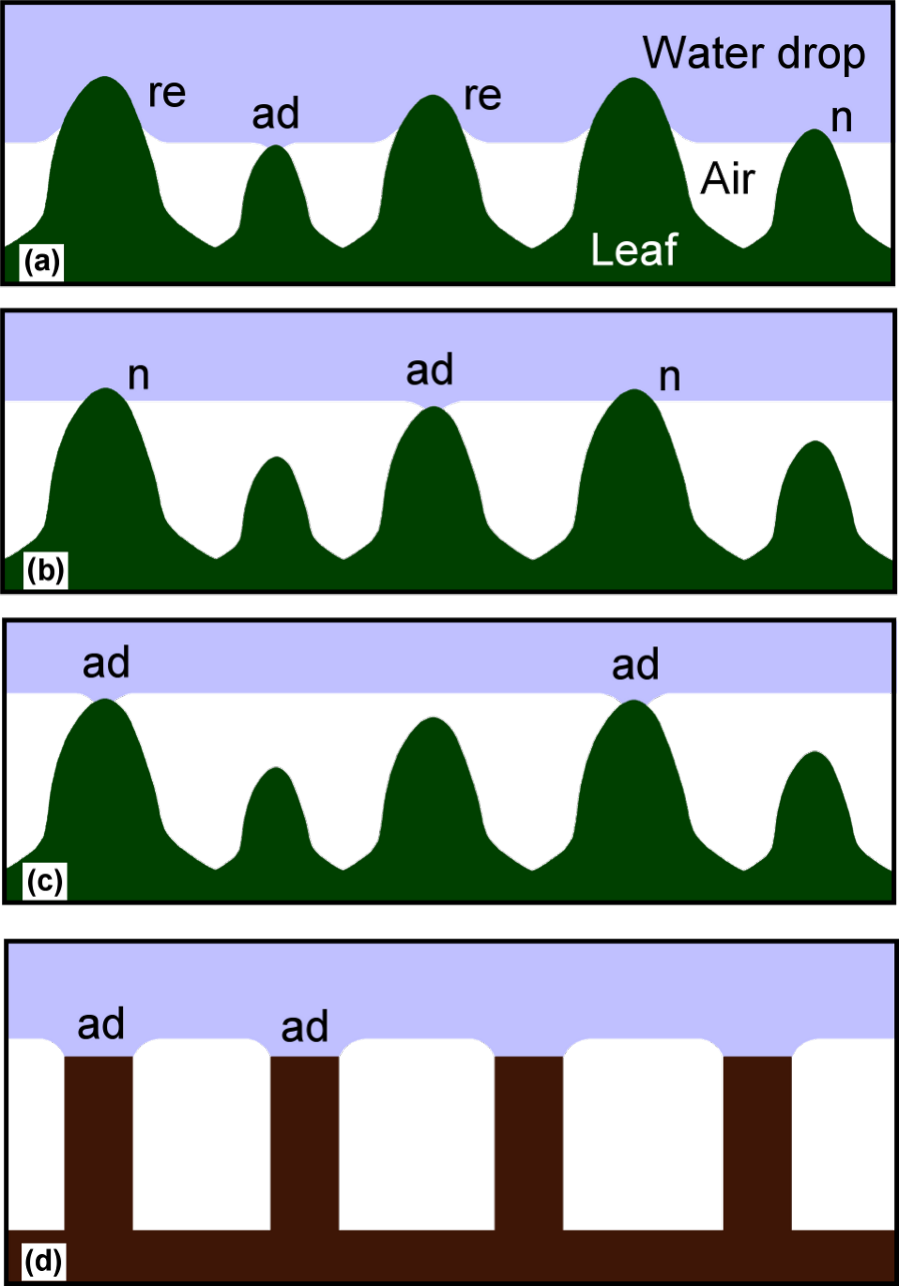
The contact between water and superhydrophobic papillae at different pressures. At moderate pressures the water intrudes into the space between the papillae, but an air layer remains between water and epidermis cells (a). The superhydrophobic surface of the papillae causes a repellent force (‘re’). When the water recedes, then the papillae lose contact one after the other (b, c). At a certain water level, the meniscus is flat and the force is neutral (‘n’). Just prior to the separation an adhesive force (‘ad’) arises at the almost horizontal area of the papilla tip, which is small on tips with intact wax crystals and larger when the wax is damaged or eroded. On artificial superhydrophobic structures with equal height (d) the adhesive forces during water receding occur simultaneously at all contacts.

**Figure 5:**
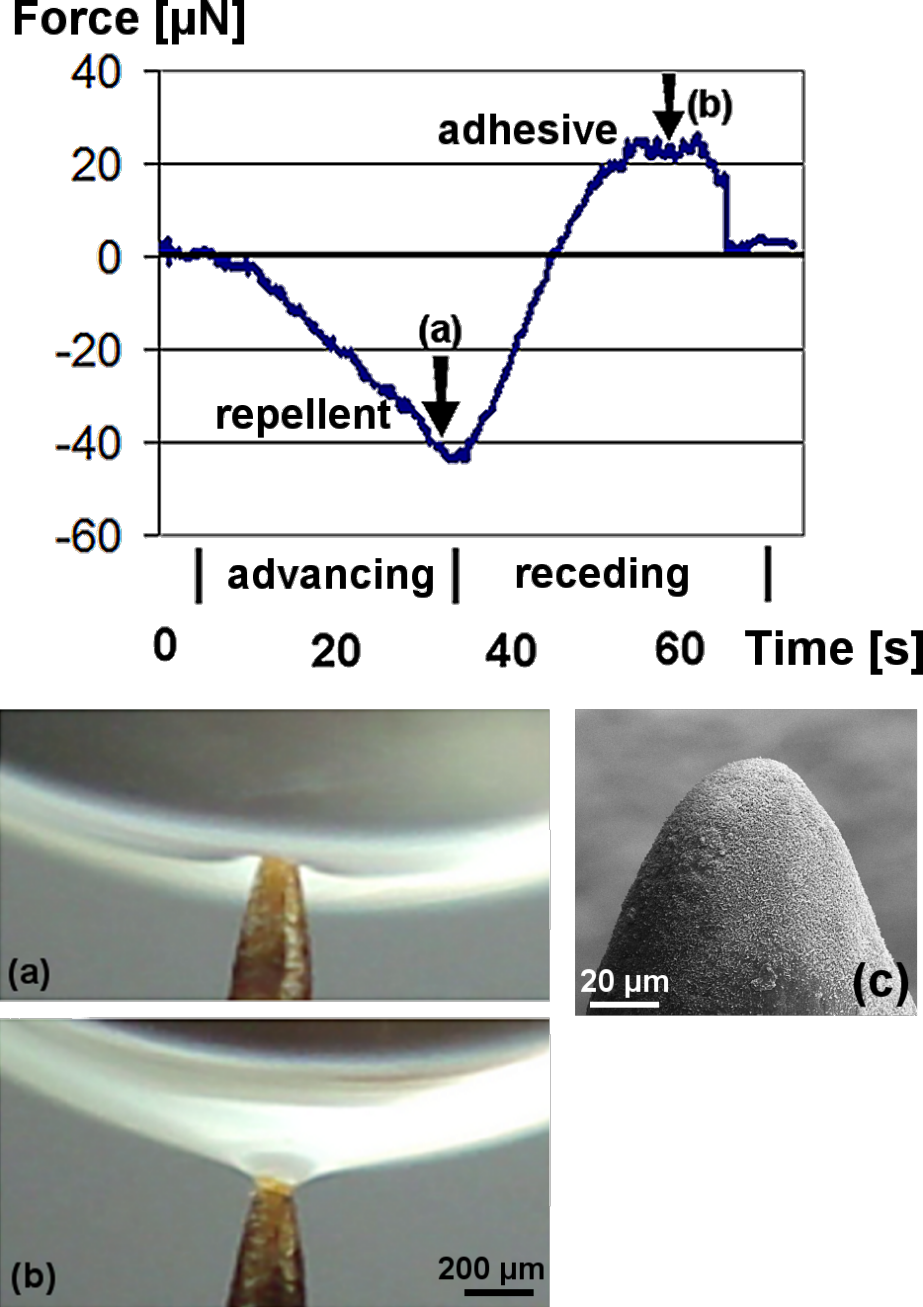
Measured forces between a superhydrophobic papilla-model and a water drop during advancing and receding. The images corresponding to the marks (arrows) in the diagram show the repellent (a) and adhesive (b) meniscus. (c) Papilla-model tip shown with SEM.

The varying height of the papillae further reduces the adhesion between water drops and the surface (Figure 4). Small resting or sliding water drops touch only the highest papillae [12]. At higher pressures, e.g., at the impact of raindrops, the water intrudes deeper between the papillae (Figure 4a) and forms a meniscus at the still superhydrophobic wax tubules coating. The deformation of the non-wetting droplet surface due to surface tension causes a repellent force (‘re’, Figure 4). When the water retracts, either at the receding side of a moving drop or if the drop is lifted off the surface, the contact areas decrease and the papillae release their contact to the water one by one (Figure 4b, Figure 4c), so that only few of the papillae are simultaneously in the adhesive state (‘ad’). Finally, before the drop loses contact with the leaf, only few of the papillae are still in contact and cause a small adhesive force. In contrast, artificial superhydrophobic samples with pillars of equal height lead to stronger adhesion during drop retraction when all the pillars are simultaneously in the adhesive state before the contact breaks (Figure 4d). The measurement of the adhesive and repellent forces between a superhydrophobic papilla-model (with ten times larger tip radius than a lotus papilla) and a water drop is shown in Figure 5.

Contact angle measurements are the standard tool for the determination of hydrophobicity. But the measurement of very high contact angles is often inaccurate due to difficulties in the determination of the exact drop shape [16], particularly on uneven leaf surfaces. For many superhydrophobic plant surfaces, the contact angles are very close together [7] such that the inaccuracies are larger than the differences between the samples. This may prevent a meaningful comparison. A more differentiated comparison of water repellency has been achieved by the measurement of the adhesion between surface and water during retraction of a drop [13], similar to the measurement shown in Figure 5. Table 1 shows, in addition to other relevant properties, the maximal adhesion forces of water drops on fresh lotus leaves and leaves of other species with intact wax. The adhesion forces are strongly dependent on surface defects which cause pinning of the drops. In contrast, advancing contact angles depend weakly on such irregularities. Thus, the adhesion data correlate better with receding contact angles and hysteresis and indicate the perfection and defects of superhydrophobic surfaces.

**Table 1:** Comparison of water repellency relevant properties of lotus and other selected species.

	*Nelumbo nucifera* (Lotus) (upper side)	*Colocasia esculenta* (upper side)	*Euphorbia myrsinites* (upper side)	*Alocasia macrorrhiza* (lower side)	*Brassica oleracea* (upper side)

papillae density (per mm^2^) [9]	3431	2662	1265	2002	0
contact angle (static) [7]	163°	165°	162°	157°	161°
drop adhesion force (µN)^a^	8–18	28–55	30–58	90–127	7–48
wax type	tubules	platelets	platelets	film on cuticular folds	rodlets and tubules
wax melting point (°C)	90–95	75–78	75–76	n.a.^b^	65–67
main components [11]	C_29_-diols	C_28_-1-ol	C_26_-1-ol	n.a.^b^	C_29_-ketones, C_29_-alkanes

^a^provided by D. Mohr, Nees Institute, Bonn; ^b^the wax film of *A. macrorrhiza* has not been isolated and analyzed; no data available.

**Figure 6:**
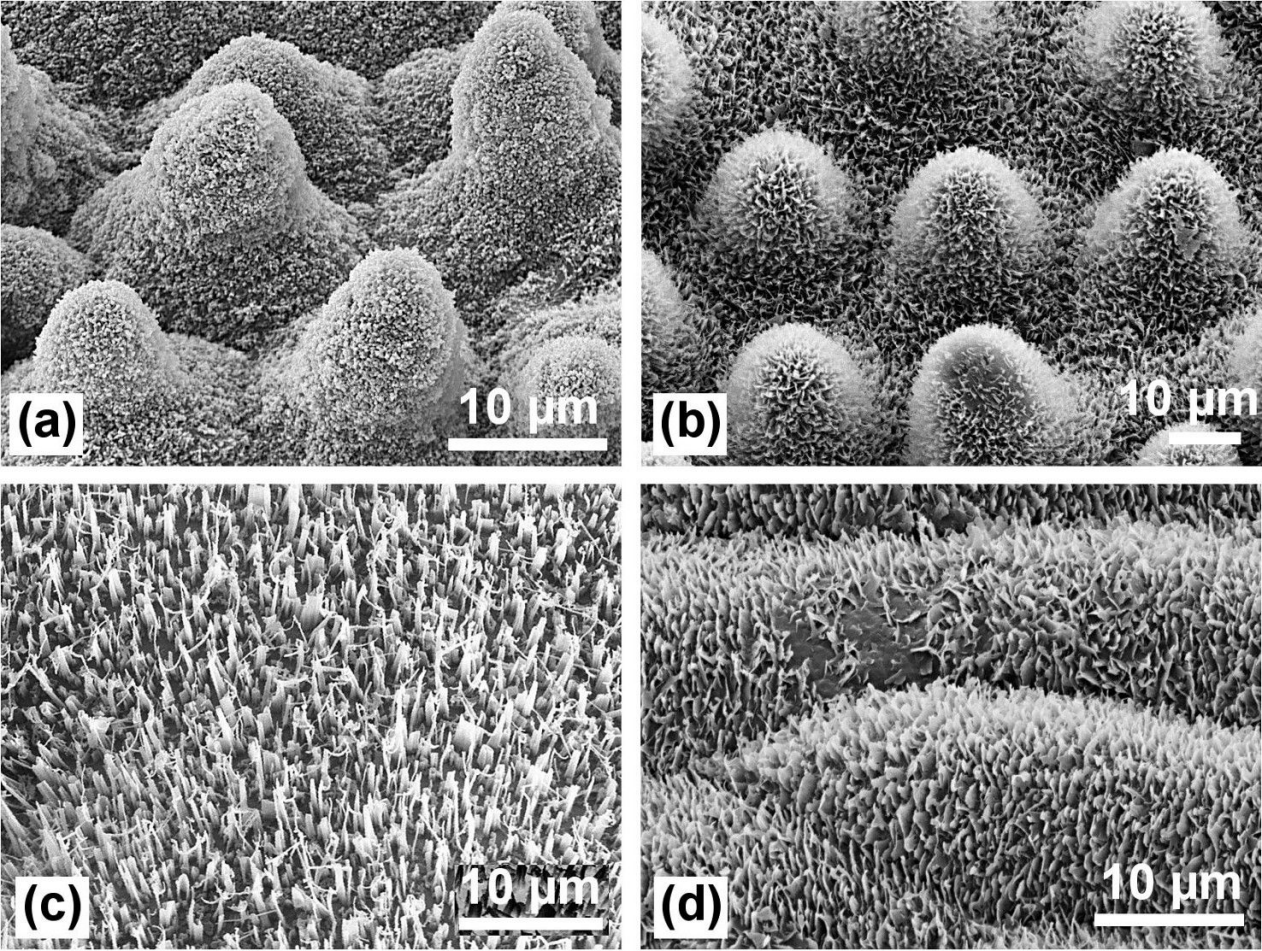
Papillose and non-papillose leaf surfaces with an intact coating of wax crystals: (a) *Nelumbo nucifera* (Lotus); (b) *Euphorbia myrsinites*; (c) *Brassica oleracea*; (d) *Yucca filamentosa*. Even the non-papillose leaves are superhydrophobic. The contact angle of *B. oleracea* can exceed 160°.

**Figure 7:**
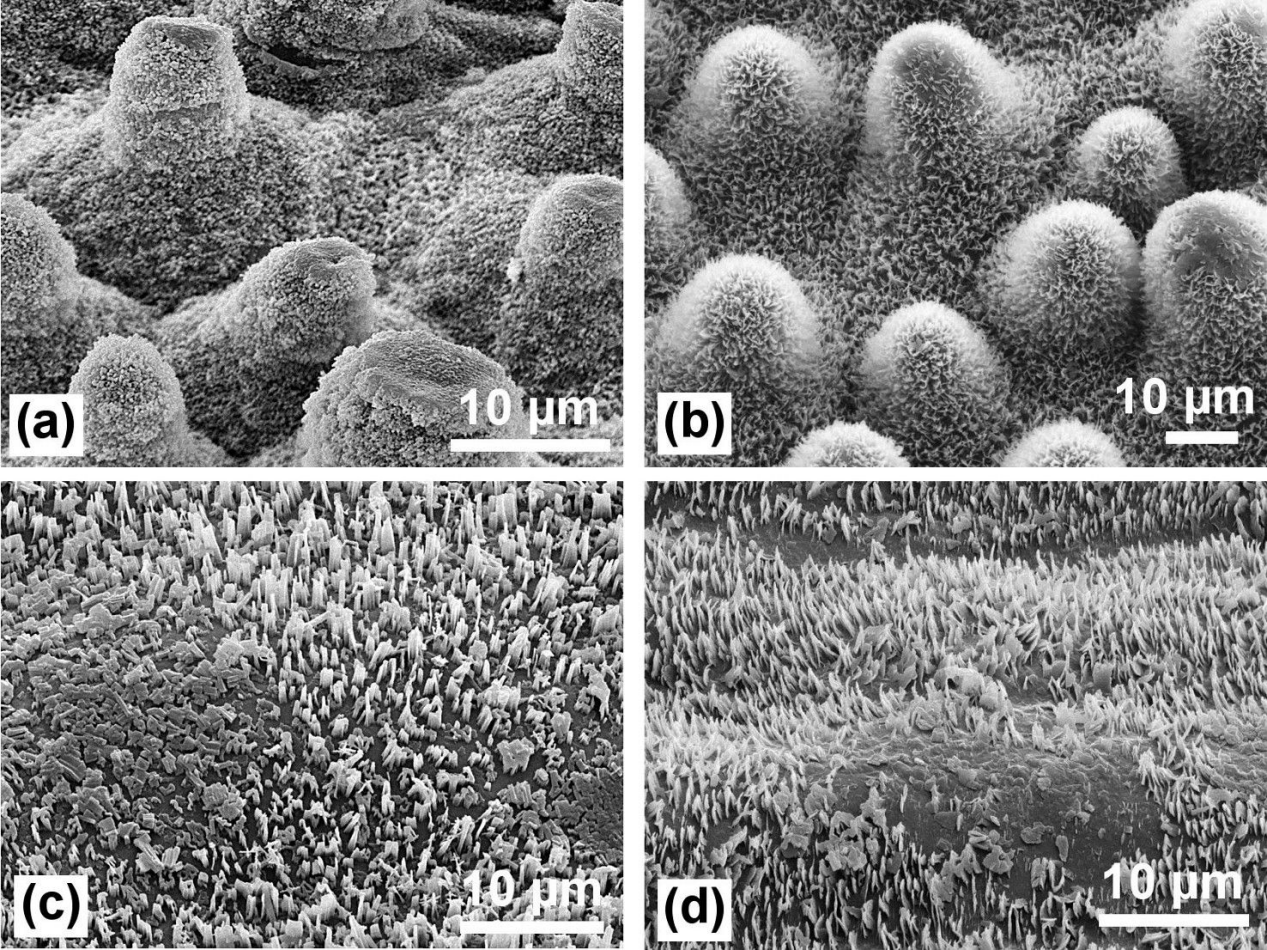
Traces of natural erosion of the waxes on the same leaves as in Figure 6: (a) *Nelumbo nucifera* (Lotus); (b) *Euphorbia myrsinites*; (c) *Brassica oleracea*; (d) *Yucca filamentosa*. On the papillose leaves (a,b) the eroded areas are limited to the tips of the papillae. On non-papillose cells, the damaged areas can be much larger (c,d), causing stronger pinning of water droplets.

**Figure 8:**
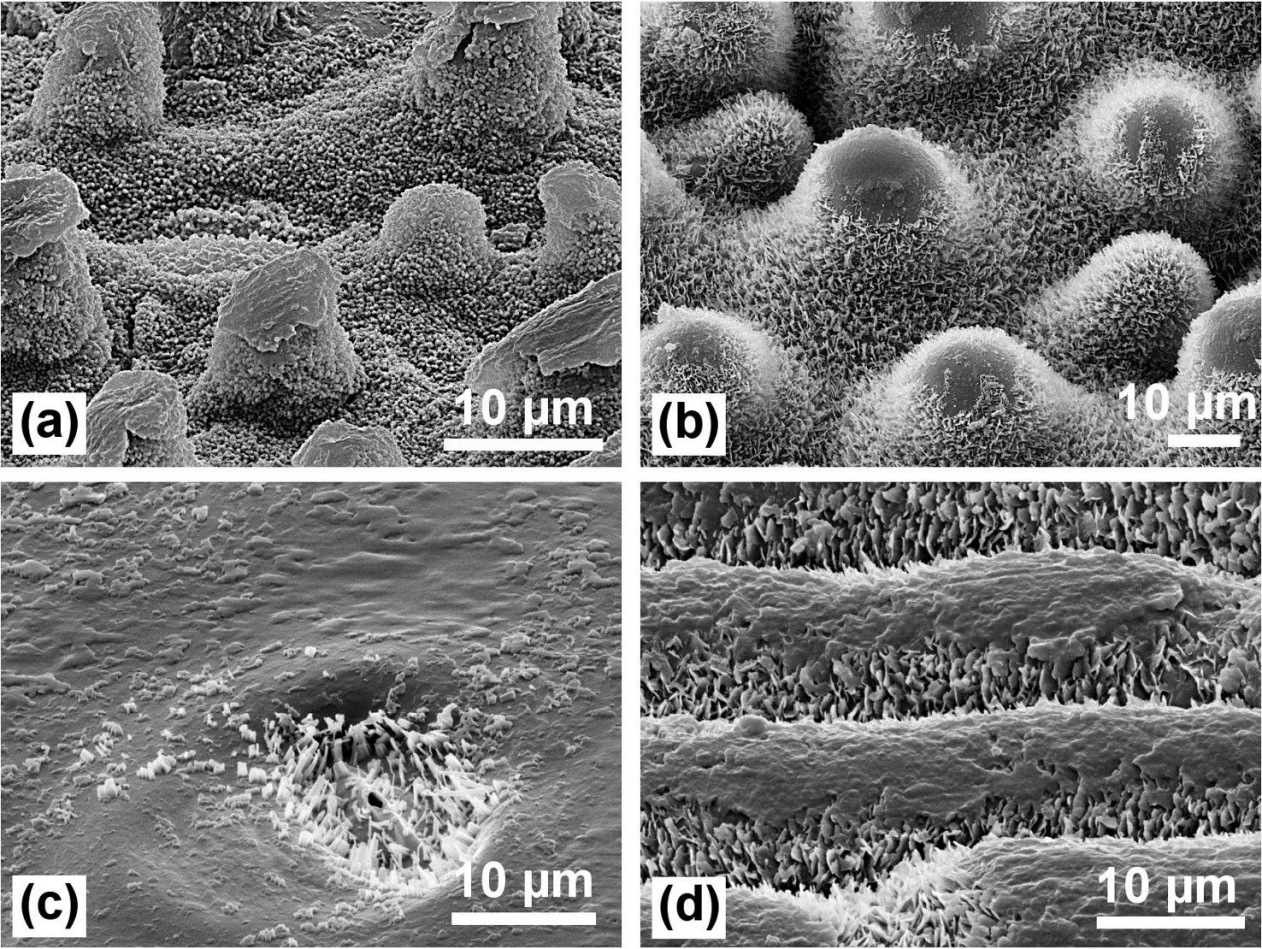
Test for the stability of the waxes against damaging by wiping on the same leaves: (a) *Nelumbo nucifera* (Lotus); (b) *Euphorbia myrsinites*; (c) *Brassica oleracea*; (d) *Yucca filamentosa*. On the papillose surfaces only the waxes on the tips of the papillae are destroyed. The waxes between the papillae are protected and remain intact. On the non-papillose surfaces, most of the waxes are destroyed, adhesion of water drops (pinning) is strongly increased, and the superhydrophobicity is lost.

**Mechanical protection of the wax crystals by papillae:** The highest water repellency occurs when the water drops touch the tips of the epicuticular wax crystals only. Thus, the best properties are found on leaves with an intact coating of wax crystals on the epidermal cells (Figure 6). The waxes are, however, relatively soft materials so that older leaves often show patches of eroded or damaged wax (Figure 7), which cause an increased adhesion of water. Neinhuis and Barthlott (1997) [7] have reported that papillae protect the wax crystals between them. On papillose epidermis cells only the wax on the papillae tips appears damaged while the wax between the papillae remains intact (Figure 7a, Figure 7b). Thus, lotus leaves retain their water repellency up to the end of their lifetime. In contrast, the non-papillose surfaces of *Brassica oleracea* and *Yucca filamentosa* (Figure 7c, Figure 7d) often show larger damaged areas which cause a stronger pinning of water. The efficiency of the protective properties can easily be tested by wiping across the leaf with the finger, which destroys only the wax on the papillae tips (Figure 8a, Figure 8b), but the leaves remained superhydrophobic. In the case of the non-papillose surface of a *B. oleracea* leaf (Figure 8c), the waxes are completely destroyed and superhydrophobicity is lost; the contact angle decreased from 160° to ca. 130°. On a *Y. filamentosa* leaf (Figure 8d) with convex epidermis cells, most of the wax crystals were destroyed and the contact angle dropped from 150° to ca. 110°.

**Figure 9:**
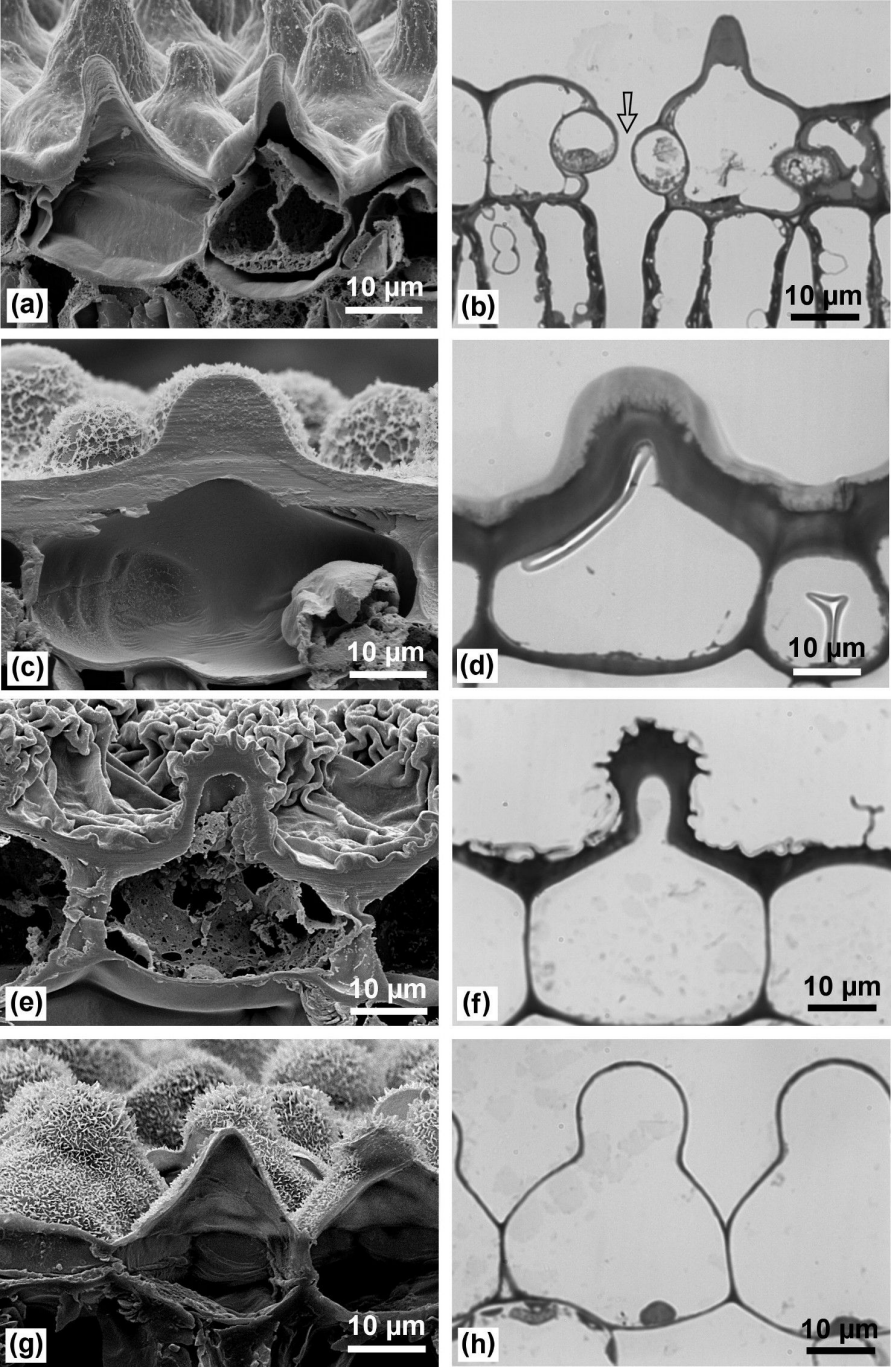
SEM and LM images of cross sections through the papillae. Lotus (a,b) and *Euphorbia myrsinites* (c,d) have almost massive papillae, those of *Alocasia macrorrhiza* (e,f) have a relatively thick outer wall; the epidermal cells of *Colocasia esculenta* have thin walls (g,h). The arrow in (b) marks a stoma.

The basis for the ability to protect the leaf surface in lotus is the robustness of its leaf papillae in combination with their high density. Cross sections (Figure 9) show that they are almost massive at least in the apical part, in contrast to the fragile papillose cells found on many flower petals. However, papillae of other superhydrophobic leaves show various architectures: *Euphorbia myrsinites* has completely massive papillae; those of the lower epidermis of *Alocasia macrorrhiza* have quite thick outer walls, whereas the epidermal cells of *Colocasia esculenta* have very thin walls with slight thickening at the protrusions.

### Properties of the lotus wax

**Figure 10:**
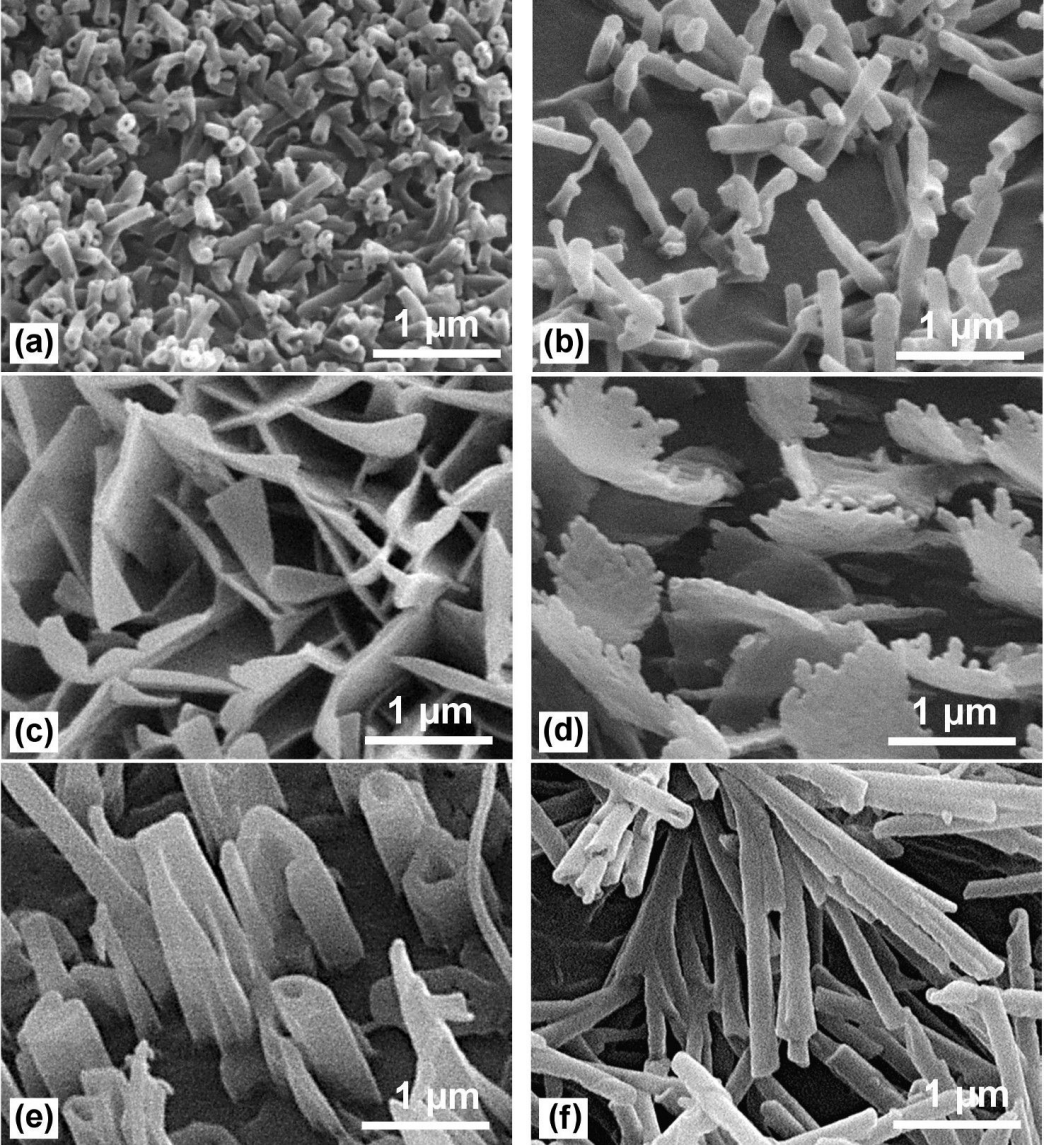
Epicuticular wax crystals in an area of 4 × 3 µm^2^. The upper side of the lotus leaf (a) has the highest crystal density (number per area) of wax crystals and the smallest spacings between them. Lotus upper side (a) ca. 200 tubules per 10 µm^2^; (b) Lotus underside ca. 63 tubules per 10 µm^2^; (c) *Euphorbia myrsinites* ca. 50 platelets per 10 µm^2^; (d) *Yucca filamentosa* ca. 17 platelets with over 80 jags per 10 µm^2^; (e) *Brassica oleracea* ca. 22 rodlets and tubules, and (f) *Eucalyptus macrocarpa* ca. 50 tubules per 10 µm^2^. The larger spacing between the wax crystals of the other surfaces compared to the lotus upper side is obvious.

Both the upper side and the lower side of the lotus leaf are covered with wax tubules. But, as can be seen on the SEM images (Figure 10a, Figure 10b), the waxes of both sides look quite different. The wax tubules of the lower side are longer (1 to 2 μm) and thicker (ca. 150 nm) and are typical ‘nonacosanol tubules’ which commonly occur on many plant species [7]. In contrast, the wax tubules of the upper leaf side are very short (0.3–1 µm) and thin (80–120 nm) but the density is very high. Figure 10 shows on a clearly arranged area, approximately 200 tubules per 10 µm^2^ on the upper side, but only about 63 tubules per 10 µm^2^ on the lower side of the same leaf. The spacing between the tubules on the upper side of the lotus leaf is much smaller than that of other wax crystals such as platelets (Figure 10c, Figure 10d) and other tubular waxes (Figure 10b, Figure 10e, Figure 10f). These distances between the hydrophobic wax crystals determine the pressure (capillary pressure) which is necessary for an intrusion of a water droplet between them.

**Figure 11:**
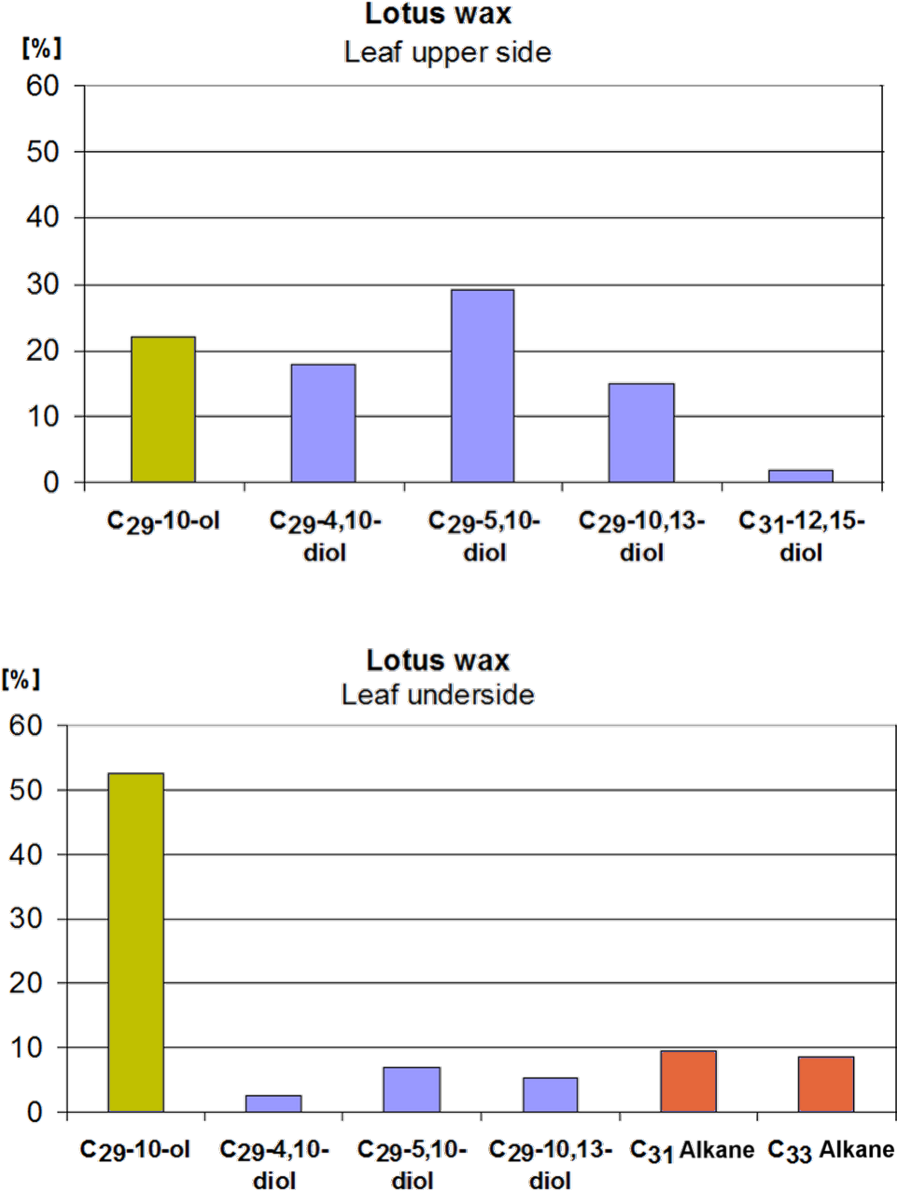
Chemical composition of the separated waxes of the upper and lower side of the lotus leaf. The upper side wax contains 65% of various diols and only 22% of nonacosan-10-ol (C_29_-10-ol), 13% was unidentified; the underside wax contains 53% nonacosan-10-ol and only 15% of various diols. Alkanes (18%) were only found in the underside wax and may be an essential part of the underlying wax film.

The chemical analyses of the waxes give an explanation for the different properties. It is known that the epicuticular wax of lotus contains a high percentage of nonacosanediols [10], but the older analyses were made from the entire wax of the leaves, which was obtained as a chloroform extract and also contained intracuticular lipids. The new analyses of the separately isolated waxes from both sides (Figure 11) show that the wax of the upper side contains ca. 65% of various nonacosanediols and only 22% of nonacosan-10-ol, whereas the wax of the underside contains predominantly nonacosan-10-ol (53%) and only 15% of diols, together with 18% of alkanes. The remaining 13% and 14% could not be identified.

**Figure 12:**
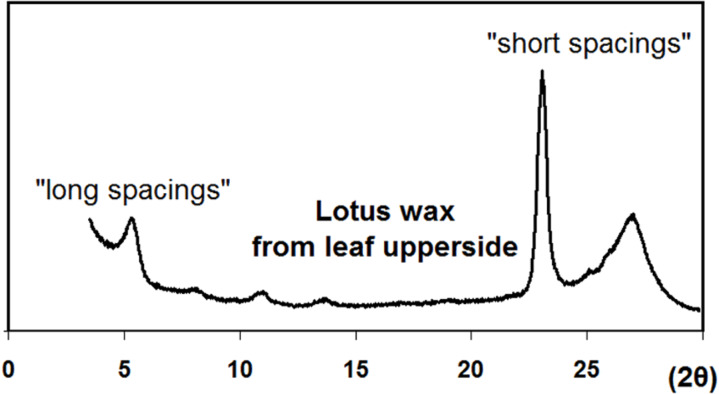
X-ray diffraction diagram of upperside lotus wax. The ‘long spacing’ peaks indicate a layer structure which is common in aliphatic waxes. The broad ‘short spacing’ peak at 2θ = 27° indicates a strong disorder in the lateral package of the molecules.

This high content of nonacosanediols provides extraordinary properties to the upper side wax. The melting point of 90 to 95 °C is very high for normal (aliphatic) waxes and indicates the influence of hydrogen bonding in the crystal lattice which increases the stability. A comparison of different aliphatic wax components with similar chain length shows that the melting points increase with the occurrence of polar OH-groups. Strong hydrogen bonding effects have been measured recently by Coward (2010) [17] in nonacosanol wax using FTIR spectroscopy. The effects on the crystal structure should be even stronger for the nonacosanediols. Although the secondary alcohols (nonacosan-10-ol and nonacosanediols) contain polar OH-groups in their molecules, the resulting wax tubules are known to feature strong and relatively stable water repellency, particularly the diols of the lotus leaf. This seems paradoxical, but X-ray diffraction analyses (Figure 12) are in accordance with a layer structure model in which the OH-groups are buried deep in the layer, while the layer surface consists only of non-polar methyl groups [11,18]. In contrast, primary alcohols such as the widespread octacosan-1-ol, which occurs in many platelet-shaped epicuticular waxes, can present the OH-group on the surface, e.g., if they are in contact with a polar environment (water). Holloway (1969) [19] studied the hydrophobicity and water contact angles of various plant waxes and pure wax components. He found the highest contact angles for aliphatic waxes which present only methyl groups on the surface.

**Figure 13:**
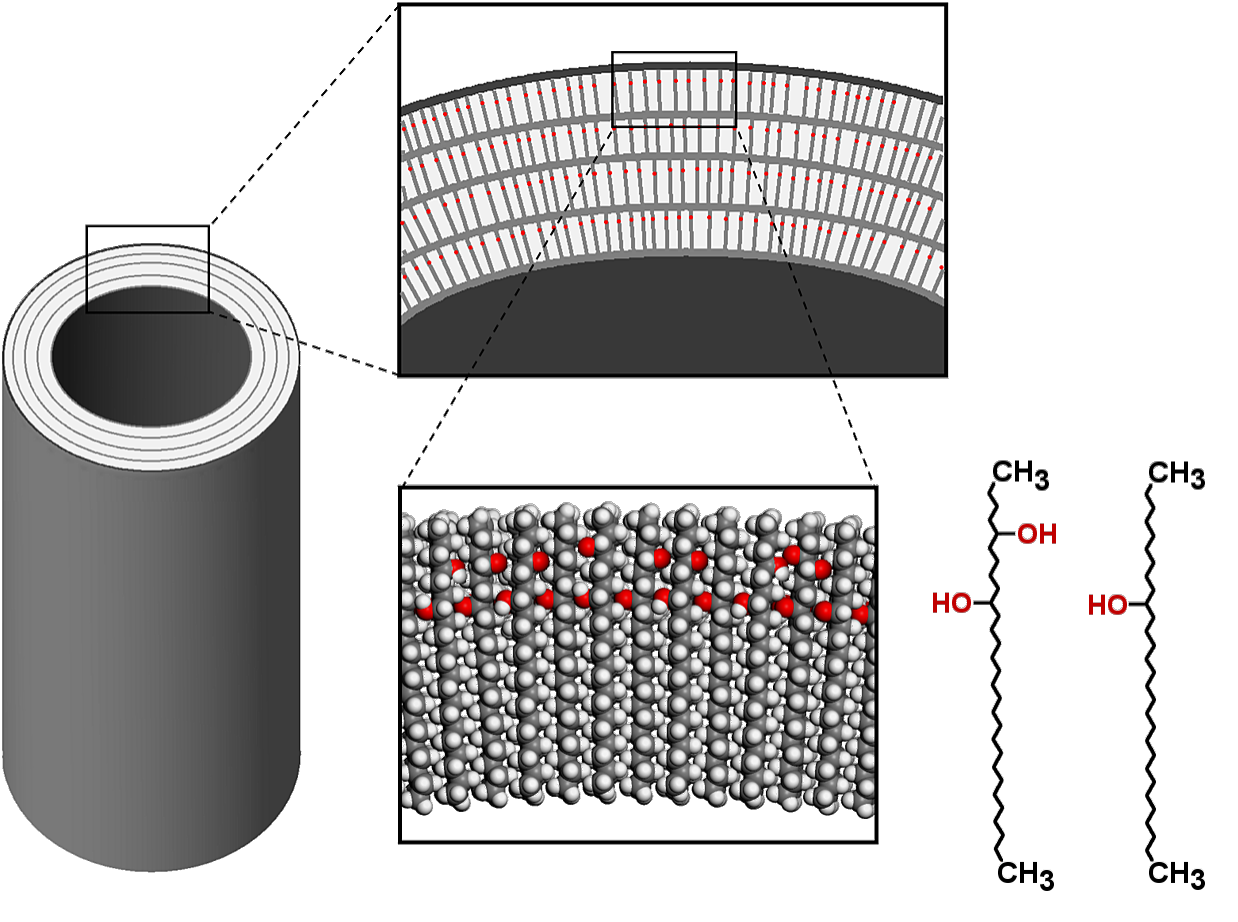
Model of a wax tubule composed of layers of nonacosan-10-ol and nonacosanediol molecules. The OH-groups (red) occupy additional space so that the dense package is disturbed and the layer is forced into a curvature which leads to the formation of a tubule. The polar OH-groups are hidden in the layer, only the CH_3_-groups appear at the surface of the layers and tubules.

According to the layer structure model, the tubules are strongly curved helically growing layers. While straight long-chained alkane molecules form flat layers and regular platelet crystals, secondary alcohols and ketones carry lateral oxygen atoms which inhibit a tight package of the molecules. Thus the resulting layers have a strong curvature and form tubules with a circular cross-section (Figure 13). Today, the progress in molecular dynamics simulations enables the calculation of the behaviour of nano-structured surfaces in contact with water [20] and to prove the theories such as those of Wenzel or Cassie and Baxter. For precise modelling of the behaviour of natural water repellent surfaces, an exact knowledge of the chemical composition and molecular structure are essential.

### Resistance against environmental stress

The excellent superhydrophobic properties of the upper side of the lotus leaf are a result of several unique optimizations. The question then arises whether this development has a certain reason or whether it is a ‘freak of nature’. On most plants, the undersides of the leaves show the highest water repellency, or more precisely, those sides which are equipped with stomata. It is obvious that the water repellency serves as a protection to keep the stomata dry [7]. On some species only the cells around the stomata are covered with wax crystals. This is in accordance with the fact that the lotus leaf is epistomatic; it bears the stomata on the upper side, which possesses the higher water repellency. The upper side of a leaf is strongly exposed to environmental impacts such as rainfall and deposition of contaminations. Obviously it is a greater challenge to keep the upper side of a large leaf dry and clean than the underside or the surfaces of vertically growing leaves (grasses etc.). On most plants, the upper sides of the leaves bear no stomata and are more robust than the undersides [21]. Thus the extremely stable and durable water repellency of the lotus leaf, which persists up to the end of its lifetime in autumn, seems to be a successful evolutionary adaptation to the aquatic environment, which led to the placing of the stomata in the upper epidermis and the development of an effective protection through specialized epidermal structures.

For a stable superhydrophobicity – that means the retention of the Cassie state with only partial contact between surface and water – an intrusion of water between the surface structures must be avoided. When the air layer is displaced by water, the water repellency is lost and the surface becomes wet (Wenzel state). The pressure which is necessary to press water into the space between hydrophobic structures depends on the local contact angle and the size of the spacing. This pressure (capillary pressure) is reciprocal to the size of the spacing and can be deduced from the Young–Laplace equation. Due to the irregular spacing, it can be estimated roughly. Water droplets with a radius <100 nm may be able to intrude between the wax tubules; this curvature corresponds to a Laplace pressure of >1.4 MPa (14 bar). Varanasi et al. (2009) [22] calculated the capillary pressures of hydrophobic test samples with structure dimensions roughly similar to those of the lotus leaf: The capillary pressure for spacing of 5 µm between hydrophobic pillars is 12 kPa (120 mbar); a nanoporous structure with 90 nm pore diameter has a capillary pressure of 1.6 MPa (16 bar). Thus the capillary pressure of the lotus papillae with spacing of ca. 10 µm is sufficient to carry the load of resting or rolling water drops. But impacting raindrops generate higher pressure pulses and can intrude into the space between the papillae. The maximal pressure for a drop impact on a rigid material can be calculated from the ‘water hammer’ equation: p_WH_ = 0.2 ρ·*c·v*, where ρ is the density of the liquid, *c* is the speed of sound in the liquid, and *v* is the velocity of the droplet. Varanasi et al. (2009) [22] calculated the ‘water hammer pressure’ of raindrops with a velocity of 3 m/s as 0.9 MPa (9 bar). However, drop impacts on flexible surfaces generate considerably lower pressures [23]. Due to the small spacing between the wax tubules of the lotus leaf and their strong hydrophobicity, their capillary pressure is obviously higher than the impact pressure of raindrops and sufficient to prevent water intrusion. However, it is unproven and hypothetical whether the larger spacing in other waxes causes an intrusion of raindrops. Mechanical damage to the waxes by the impacting drops is a more likely cause for degradation.

Biological models serve as an inspiration for the development of technical superhydrophobic materials [4]. So the question arises whether the lotus leaf presents an optimal architecture for superhydrophobicity. In biological surfaces, several different strategies can be found. The lotus leaf with the largely reduced contact area seems optimal for low adhesion of contaminants and water, observable as small roll-off angles. A disadvantage is the relatively soft wax material, which is too fragile for most technical applications. A different architecture is found on some species with hairy leaf surfaces. The water fern (some species of the genus *Salvinia*) and *Pistia stratioides* leaves retain a relatively thick air layer between hydrophobic hairs when submersed in water [24]. This provides sufficient buoyancy to avoid long-term submerging. Although superhydrophobic leaves retain an air layer when they are submersed, they are not designed for continuously living under water. All permanently submersed plant surfaces are hydrophilic without hydrophobic waxes [25]. Superhydrophobic surfaces which feature permanent air retention under water are found on animals (some birds, spiders and insects). An outstanding air-retention capability is found, for example, for the aquatic insect *Notonecta glauca* (‘backswimmer’) [26–27]. Here the water repellency is created by a two-level structure consisting of coarse hairs which can hold a relatively thick air layer, and extremely fine hairs which ensure a high capillary pressure. The biopolymers used in these structures have the advantage of a much higher strength than waxes. On the other hand, the plant surfaces have the capability to regenerate damaged or lost waxes.

## Conclusion

It is true that lotus exhibits outstanding water repellency on the upper side of its leaves. The basis of this behaviour is the hierarchical surface structure. In comparison to other species with a hierarchical surface structure composed of papillae and wax crystals, the lotus leaf shows special optimization of some of its features. The morphology of the papillae, particularly the small tip radius, minimizes the contact area to water drops but also the area where erosion and damaging of the waxes occurs. The robustness of the papillae ensures protection of the wax crystals between them. The chemical composition of the epicuticular wax with the high content of nonacosanediols leads to the growth of a dense layer of very small wax tubules with a permanently hydrophobic surface. The unique combination of these properties provides the lotus leaves with unrivaled superhydrophobicity and self-cleaning properties as an effective protection of the delicate epistomatic surface.

## Experimental

In addition to the data from the literature, some new examinations provided material for this publication. Plant leaves were taken from the Botanical Gardens, University of Bonn: *Alocasia macrorrhiza* (Elephant ear), *Brassica oleracea* var. *gongylodes* (Kohlrabi), *Colocasia esculenta* (Taro), *Euphorbia myrsinites*, *Nelumbo nucifera* (Lotus), *Yucca filamentosa*.

For scanning electron microscopy, a Cambridge Stereoscan S200 SEM was used. Depending on the sample properties, different preparation methods were applied: Slowly drying leaves were examined as fresh-hydrated samples (*Euphorbia myrsinites*, *Alocasia macrorrhiza*, *Brassica oleracea*, *Yucca filamentosa*). The other species were critical-point dried or freeze dried (Lotus). Air-dried samples were used for high-magnification imaging of epicuticular waxes. These preparation methods are described in detail elsewhere [28]. The samples for thin sections were prepared following a standard protocol for transmission electron microscopy preparation [29]: fixation in glutaraldehyde, dehydration with acetone, embedding in epoxy resin (Agar Low Viscosity Kit, Plano GmbH, Wetzlar, Germany). Sections of ca. 0.5 µm thickness were stained with ‘Rapid dye’ (Azur II and Methylene blue) for light microscopy.

Wax samples for chemical analyses were isolated mechanically using a ‘cryo-adhesion’-method using triethylene glycol as preparation liquid [30]. The wax was analysed by gas chromatography (HP 5890 series II, Avondale, USA) after ‘derivatization’ by the reaction with *N*,*O*-bis(trimethylsilyl)trifluoroacetamide [31]. X-ray powder diffraction diagrams were recorded with a diffractometer PW 1049/10 (Philips, Eindhoven, The Netherlands) [6].

Contact angles of water drops on the sample surfaces were measured with a contact angle measurement system (OCA 30-2, Dataphysics Instruments GmbH, Filderstadt, Germany) using drops of 10 µL. The adhesion of water drops on the samples was measured with a self-developed device by recording force–distance curves while the drop was attached to and detached from the surface with constant velocity. Drops of 10 µL with a diameter of 2.5 mm were attached until the contact area was 0.7 mm in diameter. Then the maximal adhesion forces during retraction were measured and compared. Low adhesion forces correlate with strong water repellency. The robustness of the leaf surface structures was tested by wiping the leaves with a finger, with a vertical force of 1 N and a contact area of 2.5 cm^2^.
